# Dermoscopic Features of Acute, Subacute, Chronic and Intermittent Subtypes of Cutaneous Lupus Erythematosus in Caucasians

**DOI:** 10.3390/jcm11144088

**Published:** 2022-07-14

**Authors:** Magdalena Żychowska, Adam Reich

**Affiliations:** Department of Dermatology, Institute of Medical Sciences, Medical College of Rzeszow University, 35-959 Rzeszow, Poland; adi_medicalis@go2.pl

**Keywords:** dermoscopy, discoid lupus erythematosus, cutaneous lupus erythematosus, lupus tumidus, acute cutaneous lupus erythematosus, subacute cutaneous lupus erythematosus, DLE, CLE, ACLE, SCLE

## Abstract

Cutaneous lupus erythematosus (CLE) is divided into the following four clinical subtypes: acute CLE (ACLE), subacute (SCLE), chronic CLE (CCLE) and lupus erythematosus tumidus (LET). The aim of this study was to describe the dermoscopic patterns of CLE by clinical variant. A total of 54 Caucasian patients from Poland (ACLE = 10; SCLE = 11; CCLE = 26; LET = 7) were included. The predefined parameters for dermoscopic assessment in inflammatory dermatoses were analyzed separately by two dermatologists. Under dermoscopy, all the variants of CLE showed predominantly polymorphous vessels on a pink–red background within the lesional skin. Dotted vessels, in association with other vessel morphologies, were observed more frequently in SCLE than in the other subtypes of CLE, but the difference did not reach statistical significance (*p* = 0.07). The findings associated with hair follicles, including rosettes (*p* = 0.02), follicular plugs (*p* = 0.01), follicular red dots (*p* < 0.01), perifollicular white halos (*p* < 0.01) and dermoscopic features corresponding to scarring, including white (*p* = 0.01) and pink (*p* < 0.01) structureless areas, were significantly more common in CCLE than in other variants of CLE. A lack of scaling, pigmentation, erosions and crusting were observed in all the cases of LET. The role of dermoscopy as an auxiliary tool in the differential diagnosis of CLE needs further elucidation.

## 1. Introduction

Skin represents the most commonly affected organ during the course of lupus erythematosus (LE). LE-specific skin lesions are referred to as cutaneous LE (CLE), and, according to the Duesseldorf classification, are divided into the following four clinical subtypes: acute CLE (ACLE), subacute (SCLE), chronic CLE (CCLE) and intermittent CLE (ICLE)—otherwise known as lupus erythematosus tumidus (LET) [1].

ACLE is a common manifestation of active systemic lupus erythematosus (SLE). It may present with a localized form (malar rash), an acute generalized form or with toxic epidermal, necrolysis-like lesions. SCLE is characterized by the presence of skin changes of annular polycyclic or papulosquamous morphology. Systemic involvement in SCLE is relatively rare, and the prognosis is favorable. The most common variant of CCLE is discoid lupus erythematosus (DLE), which typically presents with erythematous scaly lesions with follicular plugging and a tendency for central scarring. LET is an uncommon variant of CLE, which is characterized by erythematous urticarial plaques of benign and intermittent courses [2].

As clinical presentations of CLE are highly variable, they may be easily confused with other entities. Generalized ACLE may mimic contact dermatitis, atopic dermatitis, drug-induced eruption or viral exanthem [3]. In cases of SCLE, the main differential diagnoses include psoriasis, pityriasis rosea, contact dermatitis or drug-induced eruption [4]. DLE should be differentiated with cutaneous tuberculosis, lymphoproliferative disorders, sarcoidosis and, in the case of scalp involvement, lichen planopilaris. LET may show clinical similarities to polymorphic light eruptions or Jessner’s lymphocytic infiltration [5].

In addition, the individual CLE variants are not always easily distinguishable from each other. For example, early stage inflammatory DLE without marked scarring can be clinically very similar to SCLE. It should be emphasized that an accurate diagnosis is crucial, both from the point of view of therapeutic management and prognosis.

Dermoscopy is a non-invasive imaging tool that is increasingly used beyond dermatooncology in the differential diagnosis of inflammatory skin diseases. Dermoscopic findings reflect epidermal and upper-mid dermal changes and correlate directly to histopathological features [6]. Of all the clinical subtypes of CLE, the dermoscopic characteristics of CCLE, or in fact DLE, have been studied the most extensively [7,8,9,10,11,12,13,14,15,16,17,18,19,20]. On the other hand, data are scarce in the literature on the dermoscopic findings in ACLE [3,6,7,21] and SCLE [4,7,22,23]. Moreover, many of these reports concerned patients with dark skin phototypes (Fitzpatrick phototype III—V), and it remains unknown whether the results can be directly applied to the white population [3,6,7,8,9,10,11,12,13,14,15,16,20,21]. To the best of our knowledge, there are no reports in the English literature on the dermoscopic characteristics of LET.

The aim of the study was to systematically describe the dermoscopic findings in various clinical variants of CLE, namely ACLE, SCLE, CCLE and ICLE/LET.

## 2. Materials and Methods

The study was conducted between 1 December 2020 and 30 April 2022, in a tertiary dermatological center in south-eastern Poland. Patients suffering from various clinical subtypes of CLE, namely ACLE, SCLE, CCLE or ICLE/LET, were recruited for the study. All patients with ACLE fulfilled the Systemic Lupus International Collaborating Clinics’ (SLICC) criteria for SLE. The diagnosis of SCLE was based on histological and serological findings. In all cases of CCLE and LET, a histopathological confirmation of the diagnosis was available. The participants had either experienced the first episode of the disease or had presented to the department of dermatology with a significant exacerbation of the cutaneous involvement. The exclusion criteria were as follows: lack of definite diagnosis, overlap syndrome/mixed connective tissue disease or having received specific systemic or topical therapies for CLE during the previous three months.

For each participant, a target area (the most representative lesion) was selected for dermoscopic assessment. Due to their anatomic distinctiveness, CLE lesions located on the scalp or mucous membranes were not taken into consideration. All dermoscopic examinations were performed by the same dermatologist (M.Ż., the first author), using a Canfield D200^EVO^ Videodermatoscope (20–70-fold magnification). Both “dry dermoscopy” (without immersion fluid) and “wet dermoscopy” (with ultrasound gel) were performed. Minimal pressure was applied during the procedures for better visualization of vessels.

Parameters for dermoscopic assessment of inflammatory dermatoses, including morphology and distribution of vessels, color and distribution of scales, follicular findings, other structures (colors/morphologies) and presence of specific clues, were analyzed separately by two dermatologists [24]. Any discrepancies were resolved during a discussion.

The study was approved by the local ethics committee of Rzeszow University (No. 6/11/2020). All participants signed the written informed consent form prior to inclusion and consented to the publication of images.

### Statistical Analysis

Categorical data were presented as absolute numbers and percentages, and continuous data were presented as means ± standard deviation (SD) of the mean and medians (range). All statistical calculations were performed using Statistica^®^ 13.0 Software for Windows Software (Tibco, Kraków, Poland). Ch-square test, with Yate’s correction if applicable, was used for analysis of differences in the frequencies of dermoscopic findings in each subtype of CLE. A *p* value of less than 0.05 was considered to be statistically significant.

## 3. Results

A total of 54 patients were included in this study. This group consisted of 10 patients with ACLE (seven women and three men, mean age 50.3 ± 12.2 years), 11 patients with SCLE (seven women and four men, mean age 61.4 ± 14.8 years), 26 patients with CCLE (14 women and 12 men, mean age 40.6 ± 14.9 years), and seven patients with LET (three women and four men, mean age 49.7 ± 11.9 years). Among the patients with ACLE, the frequencies of the other SLICC criteria were as follows: anti-nuclear antibodies (100.0%), low complement (90.0%), leukopenia/lymphopenia (70.0%), synovitis (60.0%), non-scarring alopecia (50.0%), renal involvement (40.0%), CCLE (30.0%), hemolytic anemia (30.0%), thrombocytopenia (30.0%), an anti-dsDNA antibodies (30.0%), an anti-Sm antibodies (20.0%), anti-phospholipid antibodies (20.0%), oral ulcers (20.0%) and neurologic manifestations (10.0%). In the CCLE group, all participants were diagnosed with the discoid variant (DLE). The demographics are summarized in Table 1. Detailed dermoscopic characteristics are presented in Table 2.

### 3.1. Acute Cutaneous Lupus Erythematosus (ACLE)

**Figure 1:**
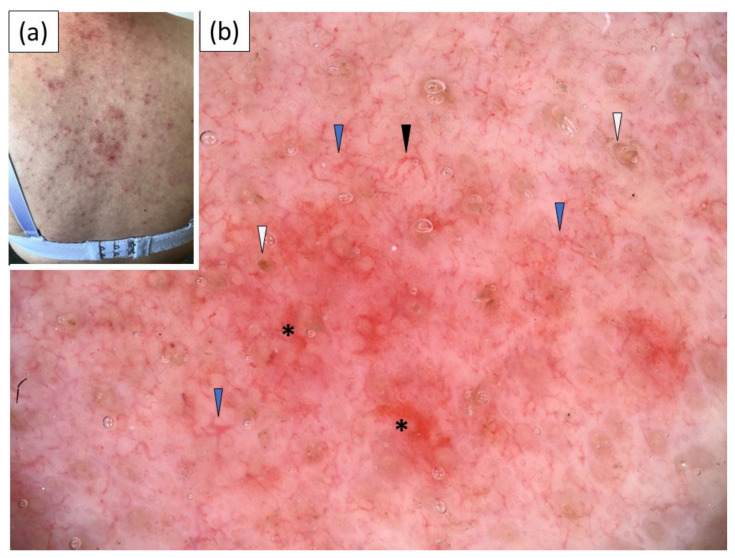
(**a**) Clinical presentation of acute cutaneous lupus erythematosus (ACLE) on the back; (**b**) videodermoscopic findings: linear (black arrowhead) and linear branched vessels (blue arrowhead), follicular plugs (white arrowhead) and red structureless (hemorrhagic) areas (black asterisk).

Under dermoscopy, all patients with ACLE showed the presence of polymorphous vessels (linear, linear branched, linear curved and, less frequently, dotted vessels) on a pink–red background. White or yellow scales of patchy distribution were observed in 30% and 20% of the cases, respectively. Follicular keratotic plugs were present in two (20%) cases, and perifollicular scaling was noted in a single (10%) case. Red structureless (hemorrhagic) areas and dotted/globular structures were present in half of the cases. Sample dermoscopic images are presented in Figure 1.

### 3.2. Subacute Cutaneous Lupus Erythematosus (SCLE)

**Figure 2:**
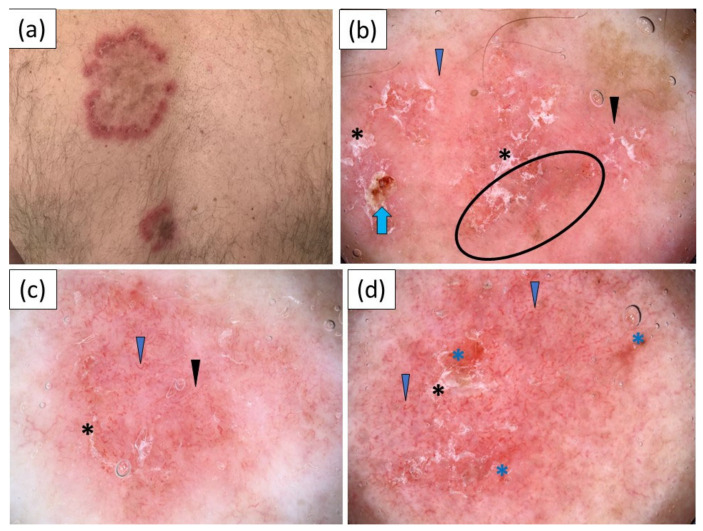
(**a**) Clinical presentation of subacute cutaneous lupus erythematosus (SCLE) on the back; (**b**–**d**) show videodermoscopic images: (**b**) videodermoscopy showing patchy white scales (black asterisk), erosion (blue arrow), scattered gray–brown dots/peppering (black circle), linear (blue arrowhead) and linear curved (black arrowhead) vessels on a pink–red background; (**c**) videodermoscopy showing discrete peripheral scaling (black asterisk) and abundance of linear (blue arrowhead) and linear branched (black arrowhead) vessels on pink background; (**d**) videodermoscopic findings: white scales (black asterisk), multiple linear, linear curved and linear branched vessels (blue arrowhead) on a pink–red background and red structureless (hemorrhagic) areas (blue asterisk).

A pink–red background and polymorphous vessels were the predominant dermoscopic findings in SCLE, noted in 90.9% and 81.8% of the cases, respectively (Figure 2). Linear, linear branched, linear curved and dotted vessels were present with comparable frequencies. White scaling was observed in the majority of the cases (63.6%). Peripheral pigmentation and red structureless (hemorrhagic) areas were noted in a significant percentage of the patients (45.5% and 36.4%, respectively). Other dermoscopic findings were less frequent.

### 3.3. Chronic Cutaneous Lupus Erythematosus (CCLE)

**Figure 3:**
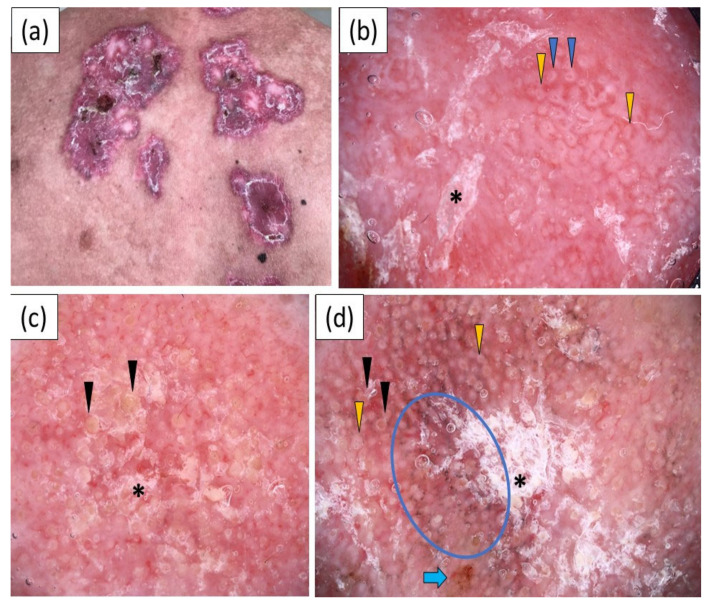
(**a**) Clinical presentation of chronic cutaneous lupus erythematosus (CCLE) on the back; (**b**–**d**) show videodermoscopic images: (**b**) videodermoscopy showing white scales (black asterisk), red follicular dots (blue arrowhead) and perifollicular white halos (yellow arrowhead); (**c**) videodermoscopy showing whitish scales (black asterisk) and follicular keratotic plugs (black arrowhead); (**d**) videodermoscopic findings: white scaling (black asterisk), follicular plugs (black arrowhead), white perifollicular halo (yellow arrowhead), gray–brown dots (blue circle) and erosion (blue arrow).

CCLE was characterized by a wide variety of dermoscopic findings (Figure 3). A polymorphous vascular pattern was present in 21 (80.8%) cases. Linear (84.6%), linear curved (80.8%) and linear branched (73.1%) vessels were predominantly observed, while dotted vessels were the least frequent vessel morphology, noted in only five (19.2%) patients. White scaling was more common than yellow scales (76.9% vs 38.5%), and it showed a predominantly patchy distribution (61.5%). Other frequent dermoscopic findings in CCLE included follicular keratotic plugs (69.2%), pink–red backgrounds (69.2%), perifollicular white halos (53.8%), dots/globules (53.8%), pink structureless areas (50.0%), red structureless (hemorrhagic) areas (50.0%), white structureless areas (46.2%), follicular red dots (38.5%), gray–brown dots/peppering (38.5%), peripheral pigmentation (38.5%), erosions (38.5%), dilated follicles (50.5%) and rosettes (30.8%).

### 3.4. Intermittent Cutaneous Lupus Erythematosus (ICLE)/Lupus Erythematosus Tumidus (LET)

**Figure 4:**
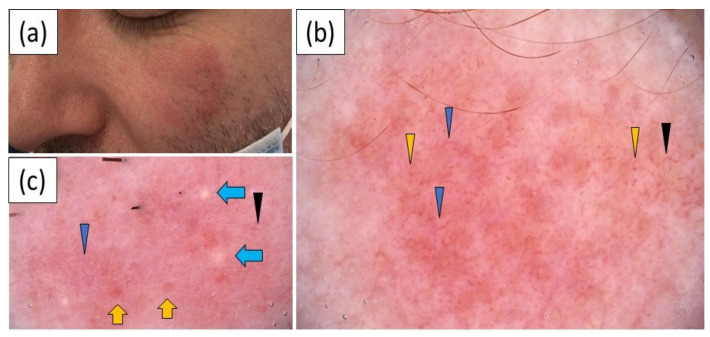
(**a**) Clinical presentation of lupus erythematosus tumidus on the left cheek; (**b**) videodermoscopy showing dotted (yellow arrowhead), linear (blue arrowhead) and linear branched (black arrowhead) vessels on a pink–red background; (**c**) videodermoscopy showing linear (blue arrowhead) and linear branched (black arrowhead) vessels, white–yellowish globules (blue arrows) and orange–reddish globules (yellow arrows).

LET was characterized by polymorphous vessels (100.0%) of unspecific distribution, (85.7%) on a pink–red background (85.7%). The vascular pattern consisted predominantly of linear (85.7%) vessels, followed by linear branched (57.1%), linear curved (57.1%) and dotted (42.9%) vessels. Follicular keratotic plugs were a relatively frequent finding, noted in three (42.9%) cases. Interestingly, the presence of scales, peripheral pigmentation, erosions and crusting were not observed in any case of LET. The dermoscopic images are presented in Figure 4.

### 3.5. Comparison of the Dermoscopic Features of ACLE, SCLE, CCLE and LET

CCLE showed some distinct dermoscopic findings. Rosettes (*p* = 0.02), follicular red dots (*p* < 0.01) and perifollicular white halos (*p* < 0.01) were only observed in CCLE. Follicular plugs (*p* = 0.01), white structureless areas (*p* = 0.01) and pink structureless areas (*p* < 0.01) were significantly more common in CCLE than in the other clinical subtypes of CLE. In addition, perifollicular pigmentation and comedo-like openings were only noted in CCLE, while perifollicular scaling and yellow structureless areas were present almost exclusively in CCLE, but the differences did not reach statistical significance (Table 2). Dotted vessels, in association with other vessel morphologies, were more frequently noted in SCLE than in the other subtypes of CLE, but the difference did not reach statistical significance (*p* = 0.07).

## 4. Discussion

The differential diagnosis of CLE can be challenging and commonly requires additional work-up and an invasive biopsy. Dermoscopy is a promising auxiliary diagnostic tool in general dermatology. There are very few reports in the literature on the dermoscopic characteristics of ACLE [3,6,7,21] and SCLE [4,7,22,23]. In generalized ACLE, Behera et al. [3] observed a multicomponent pattern, comprising white scaling, pinkish–white to reddish–white structureless areas, brown dots and globules, keratotic plugs and linear or linear branched vessels. The authors also highlighted the paucity of dotted vessels. On the other hand, a malar rash (localized ACLE) was found to predominantly present with an “inverse strawberry” pattern, consisting of reddish follicular dots, surrounded by white halos [21]. The dermoscopic findings in ACLE presumably correspond to hyperkeratosis, pigment incontinence, follicular keratotic plugs, dilated vessels and increased vasculature.

The dermoscopic characteristics of SCLE were initially described by Errichetti et al. [4], and included white scales, polymorphous vessels (linear, linear-irregular, linear branched and/or sparsely distributed dotted vessels) and pink–red backgrounds. These findings are supposed to correspond to hyperkeratosis and vasodilatation in the histopathology. In addition, the authors noted the presence of focal orange–yellowish structureless areas in 33.3% of the cases. Behera et al. [21] highlighted that dermoscopy might be useful in the monitoring of treatment efficacy in SCLE. During treatment, the authors observed the disappearance of the shiny white structures, scaling and follicular plugs, as well as a reduction in vascular structures. However, these observations were based on a single case and need further elucidation.

Several studies have focused on the use of dermoscopy in the diagnosis of DLE [8,9,10,11,12,13,14,15,16,17,18]. DLE was found to present with follicular plugs, white perifollicular halos, white scaling, speckled pigmentation, white structureless areas and linear branched vessels [8]. Nevertheless, it is worth highlighting that many of the reports concerned DLE localized on the scalp, and the anatomical distinctiveness of this location may affect the dermoscopic (trichoscopic) findings [9,10,11,12,13,14,15,16]. In addition, a substantial percentage of studies on the dermoscopy of CLE were conducted in dark-skinned individuals [3,6,7,8,9,10,11,12,13,14,15,16,20,21]. Undoubtedly, the skin color may influence the clinical and dermoscopic presentation of many entities. For example, it is believed that the gray–brown dots and globules corresponding to dermal melanin and melanophages are more commonly observed and more marked in dark-skinned individuals [3].

In the current study, we analyzed the dermoscopic features of individual clinical variants of CLE in fair-skinned patients (the white population from the Polish region), with skin lesions located beyond the scalp, to avoid the bias associated with the dermoscopic distinctiveness of this location.

A polymorphous vascular pattern on a pink–red background was a constant finding in ACLE. The vascular pattern consisted predominantly of linear, thin linear branched and/or linear curved vessels, while dotted and thick linear branched vessels were rarely present. SCLE also presented with polymorphous vessels (81.8%) and pink–red backgrounds (90.9%). However, dotted vessels, in association with linear, linear branched or linear curved vessels, were relatively common (63.6%). This dermoscopic finding distinguished SCLE from the remaining analyzed variants—ACLE, CCLE and LET—in which dotted vessels were noted in 19.9–42.9% of the cases. Although the trend of an increased frequency of dotted vessels was noted in SCLE, the difference did not reach statistical significance (*p* = 0.07). Some authors reported the presence of peripheral scaling in SCLE [21,23]. In our study, scales showed a predominantly patchy distribution (72.2%) and peripheral scaling was observed only in a single case. However, peripheral pigmentation was noted in 45.5% of the cases of SCLE, which was more frequent than in other subtypes of CLE but did not reach statistical significance (*p* = 0.14).

Of all the analyzed variants, CCLE presented with the greatest variety of dermoscopic characteristics. In particular, the dermoscopic findings associated with hair follicles were significantly more frequent in CCLE, namely rosettes (*p* = 0.02), follicular plugs (*p* = 0.01), follicular red dots (*p* < 0.01) and perifollicular white halos (*p* < 0.01). Notably, rosettes, follicular red dots, perifollicular white halos, perifollicular pigmentation, white chrysalis lines and gray–brown globules were observed exclusively in CCLE. White or pink structureless areas were also significantly more common in CCLE (*p* = 0.01 and *p* < 0.01, respectively). These dermoscopic findings typically correspond to scarring of varying degrees, therefore, it is not surprising that they were uncommonly observed in the non-scarring variants of CLE.

To the best of our knowledge, the dermoscopic pattern of LET has not been characterized yet. In our study, a polymorphous vascular pattern was a constant finding. The pink–red background was also predominantly (85.7%) present. Interestingly, follicular plugs were noted in a large percentage (42.9%) of patients. The absence of scales in all cases was noteworthy. In addition, peripheral pigmentation and yellowish crusting and erosions, which were observed with varying frequencies in other subtypes of CLE, were not present in any of the cases of LET.

A major limitation of this study was the small sample size. Therefore, variables such as the location of the lesions were not taken into consideration. The single-center design and the inclusion of only fair-skinned individuals constitute other limitations.

## 5. Conclusions

We have described the dermoscopic characteristics of individual clinical subtypes of CLE in the white population, from Poland. Polymorphous vessels on a pink–red background was the predominant dermoscopic pattern in all the variants. Dotted vessels were observed more frequently in SCLE than in the other subtypes of CLE. CCLE was characterized by a wide variety of follicular findings (rosettes, follicular plugs, red dots, perifollicular white halos, perifollicular pigmentation and perifollicular scaling) and features associated with scarring (white or pinkish structureless areas). A lack of scaling, pigmentation, erosions and crusting was typical for LET. Nevertheless, the utility of dermoscopy as an auxiliary tool in the differential diagnosis of CLE needs further elucidation.

## Figures and Tables

**Table 1:** Clinical characteristics of study participants (SD—standard deviation; CLE—cutaneous lupus erythematosus; ACLE—acute cutaneous lupus erythematosus, SCLE—subacute cutaneous lupus erythematosus; LET—lupus erythematosus tumidus).

Clinical Characteristics	CLE			
	ACLE *n* = 10	SCLE *n* = 11	CCLE *n* = 26	LET *n* = 7
Gender, *n* (%)				
Male	3 (30.0)	4 (36.4)	12 (46.2)	4 (57.1)
Female	7 (70.0)	7 (63.6)	14 (63.8)	3 (42.9)
Age, years				
Mean ± SD	50.3 ± 12.2	61.4 ± 14.8	40.6 ± 14.9	49.7 ± 11.9
Median (range)	53 (34–66)	64 (42–84)	38 (15–76)	49 (29–64)
Fitzpatrick skin phototype, *n* (%)				
I	3 (30.0)	4 (36.4)	9 (34.6)	3 (42.9)
II	6 (60.0)	7 (63.6)	13 (50.0)	4 (57.1)
III	1 (10.0)	0 (0.0)	4 (15.4)	0 (0.0)
Disease duration, months				
Mean ± SD	5.8 ± 3.5	5.9 ± 6.9	33.2 ±56	16.2 ± 20.8
Median (range)	6 (1–12)	4 (0.5–24)	11 (3–216)	9 (0.5–60)

**Table 2:** Dermoscopic findings in cutaneous lupus erythematosus (CLE), by clinical subtype (ACLE—cutaneous lupus erythematosus; SCLE—chronic cutaneous lupus erythematosus; CCLE—chronic cutaneous lupus erythematosus; LET—lupus erythematosus tumidus; *n*—number of patients).

Dermoscopic Characteristics	ACLE *n* = 10	SCLE *n* = 11	CCLE *n* = 26	LET *n* = 7	*p* Value
**Morphology of vessels, *n* (%)**					
Dotted	3 (30.0)	7 (63.6)	5 (19.2)	3 (42.9)	0.07
Linear	9 (90.0)	8 (72.7)	22 (84.6)	6 (85.7)	1.00
Linear with branches	8 (80.0)	8 (72.7)	19 (73.1)	4 (57.1)	1.00
Thick	2 (20.0)	2 (18.2)	10 (38.5)	2 (28.6)	0.55
Thin	8 (80.0)	8 (72.7)	18 (69.2)	4 (57.1)	1.00
Linear curved	7 (70.0)	8 (72.7)	21 (80.8)	4 (57.1)	1.00
Polymorphous	10 (100.0)	9 (81.8)	21 (80.8)	7 (100.0)	1.00
**Distribution of vessels, *n* (%)**					
Uniform	1 (10.0)	3 (27.3)	2 (7.7)	1 (14.3)	0.44
Clustered	0 (0.0)	1 (9.1)	0 (0.0)	0 (0.0)	0.26
Peripheral	0 (0.0)	0 (0.0)	5 (19.2)	0 (0.0)	0.11
Unspecific	8 (80.0)	7 (63.6)	17 (65.4)	6 (85.7)	1.00
**Color of scales, *n* (%)**					
White	3 (30.0)	7 (63.6)	20 (76.9)	0 (0.0)	1.00
Yellow	2 (20.0)	1 (9.1)	10 (38.5)	0 (0.0)	0.09
**Distribution of scales, *n* (%)**					
Diffuse	0 (0.0)	0 (0.0)	3 (11.5)	0 (0.0)	0.33
Central	0 (0.0)	0 (0.0)	3 (11.5)	0 (0.0)	0.33
Peripheral	0 (0.0)	1 (9.1)	5 (19.2)	0 (0.0)	0.27
Patchy	4 (40.0	8 (72.7)	16 (61.5)	0 (0.0)	1.00
**Follicular findings, *n* (%)**					
Rosettes	0 (0.0)	0 (0.0)	8 (30.8)	0 (0.0)	0.02
Follicular plugs	2 (20.0)	2 (18.2)	18 (69.2)	3 (42.9)	0.01
Follicular red dots	0 (0.0)	0 (0.0)	10 (38.5)	0 (0.0)	<0.01
Perifollicular white halo	0 (0.0)	0 (0.0)	14 (53.8)	0 (0.0)	<0.01
Perifollicular pigmentation	0 (0.0)	0 (0.0)	5 (19.2)	0 (0.0)	0.11
Perifollicular scaling	1 (10.0)	0 (0.0)	6 (23.1)	0 (0.0)	0.16
**Morphologies/colors, *n* (%)**					
White structureless areas	1 (10.0)	0 (0.0)	12 (46.2)	1 (14.3)	0.01
Pink structureless areas	0 (0.0)	1 (9.1)	13 (50.0)	0 (0.0)	<0.01
Yellow structureless areas	1 (10.0)	0 (0.0)	5 (19.2)	0 (0.0)	0.26
Dots/globules	5 (50.0)	3 (27.3)	14 (53.8)	1 (14.3)	0.18
Red globules	2 (20.0)	0 (0.0)	5 (19.2)	1 (14.3)	0.14
Gray–brown dots	1 (10.0)	2 (18.2)	10 (38.5)	0 (0.0)	0.09
Gray–brown globules	0 (0.0)	0 (0.0)	3 (11.5)	0 (0.0)	0.33
White–yellowish globules	0 (0.0)	1 (9.1)	1 (3.8)	1 (14.3)	0.57
White Lines	0 (0.0)	0 (0.0)	2 (7.7)	0 (0.0)	0.54
**Specific clues, *n* (%)**					
Peripheral pigmentation	2 (20.0)	5 (45.5)	10 (38.5)	0 (0.0)	0.14
Yellowish crust	1 (10.0)	2 (18.2)	6 (23.1)	0 (0.0)	0.47
Erosion	1 (10.0)	2 (18.2)	10 (38.5)	0 (0.0)	0.10
“sticky fiber” sign	1 (10.0)	1 (9.1)	1 (3.8)	0 (0.0)	0.76
Pink–red background	10 (100.0)	10 (90.9)	18 (69.2)	6 (85.7)	1.00
Dilated follicles	3 (30.0)	3 (27.3)	10 (38.5)	1 (14.7)	0.65
Red hemorrhagic areas	5 (50.0)	4 (36.4)	13 (50.0)	2 (28.6)	0.69
Comedo-like openings	0 (0.0)	0 (0.0)	3 (11.5)	0 (0.0)	0.33

## Data Availability

Data are available from the corresponding author upon request.
